# The Anticancer Activity for the Bumetanide-Based Analogs via Targeting the Tumor-Associated Membrane-Bound Human Carbonic Anhydrase-IX Enzyme

**DOI:** 10.3390/ph13090252

**Published:** 2020-09-18

**Authors:** Azizah M. Malebari, Tarek S. Ibrahim, Ibrahim M. Salem, Ismail Salama, Ahdab N. Khayyat, Samia M. Mostafa, Osama I. El-Sabbagh, Khaled M. Darwish

**Affiliations:** 1 Department of Pharmaceutical Chemistry, Faculty of Pharmacy, King Abdulaziz University, Jeddah 21589, Saudi Arabia; ankhayyat@kau.edu.sa; 2 Department of Pharmaceutical Organic Chemistry, Faculty of Pharmacy, Zagazig University, Zagazig 44519, Egypt; 3 Medicinal Chemistry Department, Faculty of Pharmacy, Suez Canal University, Ismailia 41522, Egypt; Dr_ibrahim_m@yahoo.com (I.M.S.); Ismail_mohamed@pharm.suez.edu.eg (I.S.); samiamostafa2010@hotmail.com (S.M.M.); Khaled_darwish@pharm.suez.edu.eg (K.M.D.); 4 Medicinal Chemistry Department, Faculty of Pharmacy, Zagazig University, Zagazig 44519, Egypt; osamaelsabbagh@yahoo.com

**Keywords:** membrane-bound human carbonic anhydrase, anticancer, bumetanide, sulfonamides, molecular docking

## Abstract

The membrane-bound human carbonic anhydrase (hCA) IX is widely recognized as a marker of tumor hypoxia and a prognostic factor within several human cancers. Being undetected in most normal tissues, hCA-IX implies the pharmacotherapeutic advent of reduced off-target adverse effects. We assessed the potential anticancer activity of bumetanide-based analogues to inhibit the hCA-IX enzymatic activity and cell proliferation of two solid cancer cell lines, namely kidney carcinoma (A-498) and bladder squamous cell carcinoma (SCaBER). Bumetanide analogues efficiently inhibit the target hCA-IX in low nanomolar activity (IC_50_ = 4.4–23.7 nM) and have an excellent selectivity profile (SI = 14.5–804) relative to the ubiquitous hCA-II isoform. Additionally, molecular docking studies provided insights into the compounds’ structure–activity relationship and preferential binding of small-sized as well as selective bulky ligands towards the hCA-IX pocket. In particular, 2,4-dihydro-1,2,4-triazole-3-thione derivative **9c** displayed pronounced hCA-IX inhibitory activity and impressive antiproliferative activity on oncogenic A-498 kidney carcinoma cells and is being considered as a promising anticancer candidate. Future studies will aim to optimize this compound to fine-tune its anticancer activity as well as explore its potential through in-vivo preclinical studies.

## 1. Introduction

As a worldwide healthcare burden, cancer has enormous financial and psychological impacts on patients, families, and healthcare systems [1]. The World Health Organization Global Cancer Report 2014 projected a 57% worldwide cancer incidence increase within the next 20 years [2]. The market-available classical or even novel targeted chemotherapeutic agents possess several adverse effects affecting patient compliance as well as increasing cancer-related drug resistance [3,4]. Resistance is currently responsible for most cancer relapses, causing up to 90% of the cancer-related mortalities [5,6,7,8,9]. All such concerns emphasize the urgent need for newer, more selective therapeutic agents that can target the cancer-related drug resistance mechanism as well. Notably, targeting the metalloenzyme carbonic anhydrases (CAs, EC 4.2.1.1) for inhibition has become an exciting and hot topic within the last decade [10,11,12].

The CAs possess significant roles, including the transport of bicarbonate/carbon dioxide, carbon dioxide/pH homeostasis, electrolyte secretions, biosynthetic reactions, and bone resorption [13,14,15,16]. The catalysis of these metalloenzymes is through a nucleophilic attack upon carbon dioxide via a zinc-bound hydroxide ion followed by catalytic site regeneration and proton dissociation, mediated by the ionization of the zinc-bound water molecule [17]. Currently, six evolutional CA families have been recognized, where only the α-class CAs are expressed in humans with a total of 16 isoforms [18]. Of the human CAs, the membrane-bound isoforms (*h*CA-IX and -XII) have great activity, have been associated with the process of tumorigenesis and cancer progression/metastasis, and considered as tumor clinical prognosticators [19,20,21,22,23,24,25,26,27]. Notably, *h*CA-IX is a significant human CA isozyme, being confined to few normal tissues (i.e., gut and lining of body cavities) [13,21]. Thus, targeting *h*CA-IX implies the pharmacotherapeutic advent of reducing unwanted off-target binding to the ubiquitous mitochondrial or cytosolic *h*CAs [28].

The high overexpression and ectopic induction of *h*CA-IX within several solid tumors recognize such an isozyme as a relevant therapeutic biotarget for aggressive tumors [29,30]. The *h*CA-IX overexpression in response to hypoxia is related to its catalytic ability to efflux acids permitting tumor cell survival, while simultaneously regulating the cancer microenvironment towards an acidic milieu promoting tumor growth, invasion, and metastasis [19,31,32,33]. Based on the above evidence, *h*CA-IX has become a validated biotarget, furnishing several anticancer therapeutic benefits, and triggering many research groups to develop *h*CA-IX inhibitors with impressive isoform specificity.

**Figure 1:**
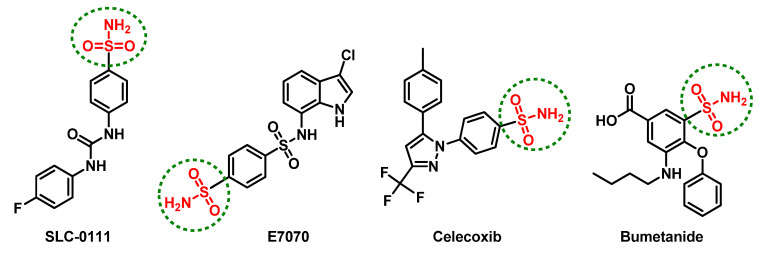
The chemical architecture of promising anticancer sulfonamides in clinical trials or currently in-use pharmacotherapeutics with inhibition activity on the tumor-associated human carbonic anhydrases (Cas).

Several promising anticancer agents have been reported incorporating the ionizable sulfonamide (-SO_2_NH-) group [34]. Such rigid functionalities serve as efficient zinc-binding groups for ligand anchoring where they can coordinate, through their negatively deprotonated forms, to the catalytic Zn(II) cation for replacing the zinc-bound water molecule and achieving a tetrahedral-coordinated geometry [35,36]. Furthermore, the hydrogen atom on the coordinated nitrogen atom satisfies the hydrogen-bond accepting character of the conserved histidine residue within the CAs active sites [37]. Both the phase-I/II clinical candidates, SLC-0111 and E7070 (Figure 1), harbor such an ionizable group and have been investigated for the management of advanced metastatic or relapsed cancers (refer ClinicalTrials.gov; NCT02215850 and NCT01692197, respectively). At the same time, several therapeutics in clinical use, such as celecoxib and bumetanide (Figure 1), possess significant inhibition activity towards the tumor-associated *h*CAs related to their sulfonamide-pharmacophoric feature, in addition to their main pharmacotherapeutic activity [38,39,40]. Bumetanide showed low nanomolar inhibition activity on the tumor-associated *h*CA isoforms (*K*i = 21.1–25.8 nM) and is a promising effective anticancer agent because it also showed moderate (250–700 nM) or weak (2.57–6.98 μM) inhibition for mitochondrial or cytosolic *h*CAs, respectively [39,41]. Such a preferential pattern may be related to the sterically hindering phenoxy moiety being *ortho* to the sulphamoyl-Zn(II)-binding group. This bulky group hinders the bumetanide anchoring within *h*CA-I- or -II-occluded active sites where respective bulky residues (His67/Phe91/Tyr204 or Phe131) are present at these site entrances [39].

**Figure 2:**
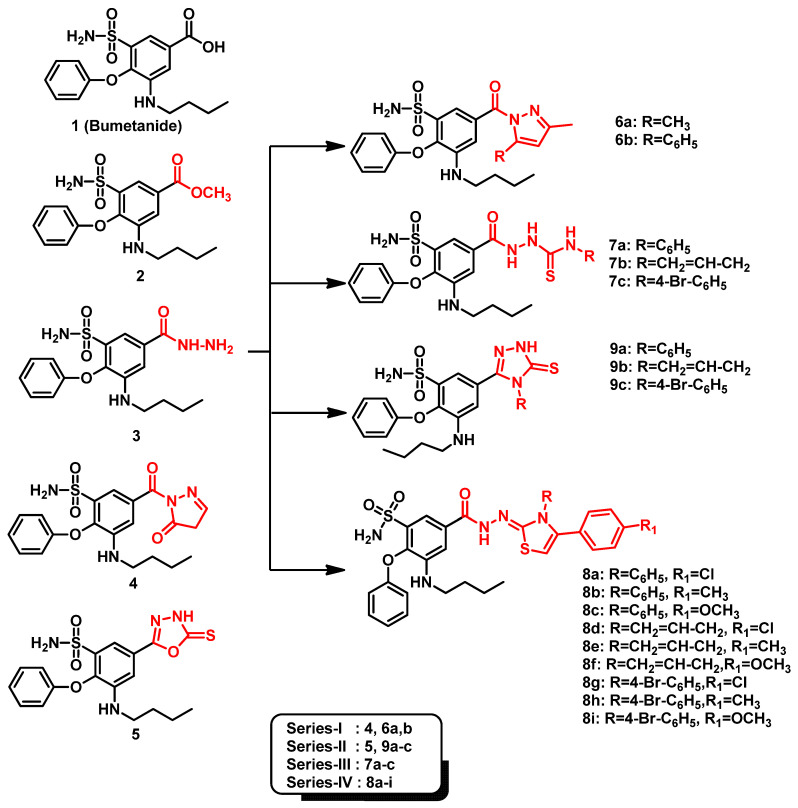
The chemical structures of the proposed benzenesulfonamide-based compounds as promising *h*CA-IX inhibitors. They are classified into four **Series** (**I**–**IV**) based on tail part homology, bioisosteric homology, and spatial size similarity.

Recently, we reported novel benzenesulfonamide compounds, based on the bumetanide core scaffold, as selective COX-2 inhibitors exhibiting promising anti-inflammatory activities superior to celecoxib (Figure 2) [42]. These compounds were synthesized through converting bumetanide into an acid hydrazide key intermediate (3) harboring the benzenesulfonamide head together with both ortho- and meta-bulky substitutions. The acid hydrazide tail, at the other meta-position, was considered as a handy functional group that was subsequently derivatized and/or structurally expanded to yield all other synthetic compounds. Condensation of the key acid hydride with ethylacetoacetate, β-diketones, acetylacetone, or benzoylacetone provided either the pyrazolone or pyrazole derivatives (**Series-I**). However, reaction with different isothiocyanates afforded the thiosemicarbazide derivatives (**Series-III**) which were further cyclized via sodium hydroxide/acetic acid or phenacyl bromide to yield the target 1,2,4-triazole (**Series-II**) or thiazole (**Series-IV**) derivatives, respectively. Here, within this manuscript, we aimed to assess the potential anticancer activity for this series of bumetanide analogues using colorimetric in-vitro bioassays relying on the biochemical blockage of the isolated *h*CA-IX enzymatic activity in addition to cell proliferation inhibition of two solid cancer cell lines (A-498/kidney carcinoma and SCaBER/bladder squamous cell carcinoma). Moreover, the impact of the selected compound upon the A-498/kidney carcinoma cell cycles was evaluated through cell cycle analysis and apoptosis rate investigations. Finally, molecular docking studies employing *h*CA-IX atomic structure (PDB ID: 3iai) [43] were conducted to unveil the key ligand–receptor interactions and deduce the structural activity relationship of these compounds.

## 2. Results and Discussion

### 2.1. Biological Evaluation

#### 2.1.1. Carbonic Anhydrase Inhibition Assay

The in-vitro human CA-II (cytosolic) and -IX (transmembrane, tumor-associated marker) inhibition assay has been estimated for the bumetanide-based analogues while assigning acetazolamide as their positive control standard (Table 1) using a colorimetric CA-inhibition assay based on the target CA-esterase activity [44,45].

Based on the furnished inhibitory data illustrated in Table 1, several structural activity relationships have been deduced. The bumetanide-containing compounds displayed weak to potent inhibitory activity toward the physiologically dominant off-target isoform *h*CA-II, with IC_50_ ranging from high nanomolar to low micromolar concentrations (IC_50_ = 205.08 and 5117 nM) with statistical significance (*p* < 0.05) as compared to reference control. The derivative of acetazolamide acted as a nanomolar *h*CA-II inhibitor with IC_50_ of 17.1 nM. The non-cyclic tailed compound (**2**) acted as a more than two-fold more potent *h*CA-II inhibitor (IC_50_ = 668.15 nM) than its carboxyhydrazine bioisostere (**3**) (IC_50_ = 1614.82 nM). Bumetanide bearing 5-thioxo-1,3,4-oxadiazole 5 (IC_50_ = 486 nM) displayed enhanced activity compared to the pyrazolone derivative **4** (IC_50_ = 1052 nM). Moreover, replacement of the carbonyl group on pyrazole **4** with the phenyl ring **6b** led to about a one-fold enhanced *h*CA-II inhibition potency. Bumetanide containing a hydrazinecarbothioamide tail in **Series III** (**7a**–**7c**) exhibited weaker inhibition activity with IC_50_ > 1500 nM, compared to the rigid triazole **Series II** (**9a**–**9c**) with IC_50_ < 500 nM, except for the phenyl ring on the triazole compound 9a (IC_50_ = 4061 nM), which displayed a higher value than its corresponding hydrazinecarbothioamide chain derivative **7a** (IC_50_ = 1571 nM). It is noteworthy that different activity profiles have been assigned to the bulky branched-tailed members of **Series IV** based on relevant substitute combinations around the compounds’ thiazoline-based trunks. Members bearing the thiazole moiety (**8a**–**8i**) showed improved activity in the submicromolar range (208–999 nM), except for the 4-chloro-phenyl-3-phenyl-thiazol derivative **8b** and the 4-chloro-phenyl-3-allyl-thiazolderivative **8d**, which displayed weak inhibition efficacy in the micromolar range against *h*CA-II. Among **Series IV**, the compounds harboring the *N*-(*p*-bromophenyl) groups (**8g**–**8i**), exhibited the best *h*CA-IX inhibition activity down to sub-micromolar activities (IC_50_ = 208.81–784.08 nM). Concerning the substitution at C4 of the thiazoline trunk, **8h** possessing the *p*-tolyl group was at higher potency (IC_50_ = 205.2 nM) compared to those with terminal chlorophenyl **8g** or methoxyphenyl groups **8i** (IC_50_ = 784.0 and 426.4 nM, respectively). Therefore, a favored substitute combination between the *N*-(*p*-bromophenyl) and 4-(*p*-tolyl) functionalities is suggested to be beneficial for obtaining potent thiazoline-based *h*CA-II inhibitors.

An observable inhibitory pattern has been deduced between the two investigated *h*CA isozymes. The tumor-associated *h*CA-IX isoform was strongly inhibited by all the examined compounds, with IC_50_ ranging between 4.4 and 23.7 nM with statistical significance (*p* < 0.05) as compared to reference control. These high-activity data came superior over several reported hCA-IX inhibitors such as the polycyclic sulfonamides (IC_50_ = 260–21.2 nM) [46] and close related aromatic sulfamates (IC_50_ = 27,719–17.6 nM) [47]. On the other hand, the presented compounds were found, to a certain degree, comparable to some members of celecoxib-mimic sulfonamides as reported hCA-IX inhibitors (IC_50_ = 640–5 nM) [48]. Nevertheless, lower off-target profile was depicted for the proposed compounds, as compared to the latter reported classes, for exhibiting much higher IC50 values (upper micromolar range) on the hCA-II enzyme.

Among the presented compounds, bumetanide methyl ester **2** was equipotent to bumetanide ~23 nM and displayed potent activity compared to acetazolamide (IC_50_ = 31.63 nM). At the same time, bumetanide hydrazide **3** emerged as the most efficient *h*CA-IX inhibitor with single-digit nanomolar activity (IC_50_ = 7.87 nM) compared to its corresponding rigid heterocyclic pyrazolone derivative 4 and 5-thioxo-1,3,4-oxadiazole derivative **5** (IC_50_ = 16.45 and 12.44 nM, respectively). Incorporation of a phenyl on the pyrazole ring of bumetanide analogue **6b** (IC_50_ = 5.43 nM) resulted in three-fold enhanced activity compared to the methyl on pyrazole **6a** (IC_50_ = 16.56 nM). The hydrazinecarbothioamide chain on the bumetanide scaffold (**7a**–**7c**) enhanced the inhibition activity against *h*CA-IX with two-digit nanomolar activity, while a remarkable improvement in *h*CA-IX inhibition potency with one digit nanomolar activity was achieved upon cyclization of compounds (**7a**–**7c**) to the triazole moiety (**9a**–**9c**). Thiazole-based compounds in **Series IV** exhibited potent inhibition activity with a range of 6.36–14.31 nM.

Introduction of an electron-withdrawing group (EWG) such as chloro-phenyl on the thiazole nucleus attached with a different substituent: phenyl **8a**, allyl **8d**, and 4-bromophenyl **8g** (IC_50_ = 6.53, 6.36, and 7.18 nM, respectively) elicited an enhancement of effectiveness against *h*CA-IX compared to electron-donating groups (EDG), such as methyl-phenyl, on thiazole compounds; phenyl **8b**, allyl **8e**, and 4-bromophenyl **8h** (IC_50_ = 12.97, 11.51, and 14.31 nM, respectively) and methoxy-phenyl thiazole analogues; phenyl **8c**, allyl **8f**, and 4-bromophenyl **8i** (IC_50_ = 12.60, 13.41, and 10.57 nM, respectively). Compound **9c** with 4-bromophenyl on triazole ring showed impressive inhibition potency, with IC_50_ = 4.42 nM, compared to its corresponding 4-bromophenyl on thizaole ring derivatives **8g**, **8h** and **8i** (IC_50_ > 7 nM). Such preferential activity suggests the influence of orientation and interactions of the *p*-bromophenyl motif on directing the orientation of the extended, tailed functionalities to achieve favored compound-target interactions within the pocket of *h*CA-IX by furnishing different contacts with the lining residues. The selectivity indexes (SI) for inhibiting the tumor-associated isoform *h*CA-IX over the off-targeted cytosolic isoform *h*CA-II have been presented in Table 1. All the tested compounds possessed excellent selectivity (SI = 14.5–804).

Interesting inhibition profiles have been reported for the bumetanide compounds correlating to their respective sizes. Notably, the small-sized ligands, as with members of both **Series I** and **II**, exhibit a better inhibitory profile towards the tumor-associated *h*CA isozyme than the bulkier-tailed compounds in **Series III**. The latter observation could be related to the lack of accommodation of the linear thiosemicarbazide tails within the *h*CA-IX pocket. Nonetheless, the *h*CA-IX isoform was depicted to be more tolerable for other bulky-tailed compounds, where several **Series IV** members exhibited sub-micromolar activities. Such favored activities suggest a different binding mode; the **Series IV** members may anchor their sterically hindered hydrazone-based extremities in a way favoring contacts with the pocket residues.

#### 2.1.2. Cell Proliferation Assay

**Figure 3:**
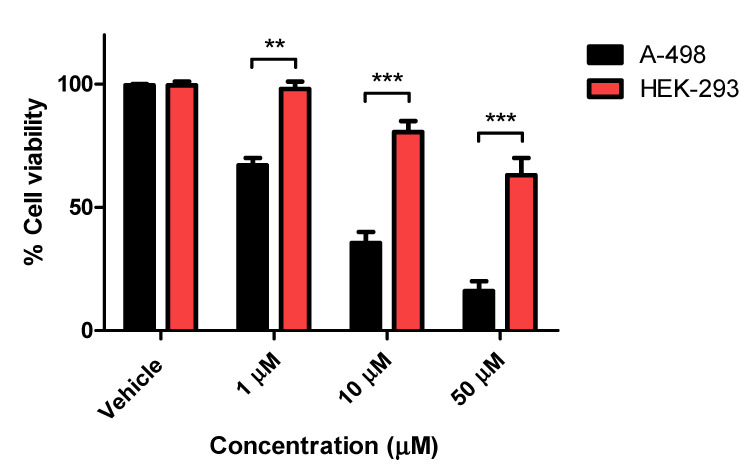
Antiproliferative activity of compound **9c** in tumorigenic A-498 cells and non-tumorigenic HEK-293 cells. The experiment was performed in triplicate and significance was represented as mean ± S.D. ** = *p* < 0.01; *** = *p* < 0.001 between indicated groups.

Compounds **8a**, **8h**, **9b**, and **9c** were selected to be investigated for their potential antiproliferative activity on two different solid cancer cell lines, A-498/kidney carcinoma and SCaBER/bladder squamous cell carcinoma, and acetazolamide as the positive control standard. As shown in Table 2, compound **9c** displayed potent antiproliferative activities against both cell lines A-498 and SCaBER with IC_50_ values of 5.07 and 8.80 µM, respectively, which is more potent than the reference drug acetazolamide (IC_50_ 40.0 and 40.6 µM, respectively) with *p* < 0.05 as compared to control. Compounds **8a** and **9b** exhibited moderate activity against the A-498 cell line with an IC_50_ value equal to 15.6 and 16.0 µM, respectively. Compound **8h** exerted impressive antiproliferative activity on A-498 cells (IC_50_ 6.55 µM) but a poor effect on SCaBER cells (IC_50_ 78.04 µM) with *p* < 0.05 as compared to control. Such differential activity patterns could be explained as off-target activity mediated by the latter compounds being related to a specific cancer cell line. The latter suggestion was reported by Mboge et al. when their substituted benzene sulfonamides were able to inhibit cell growth even within cell lines where *h*CA-IX expressions were ablated [49]. The non-tumorigenic cell line HEK-293 (normal human embryonic kidney) was chosen to investigate the toxicity and selectivity of the most potent compound **9c**. As shown in Figure 3, the IC_50_ value of **9c** was greater than 50 µM in HEK-293 cells which was significantly higher than that observed against the A-498 and SCaBER9 cancer cell lines (IC_50_ = 5.07 µM and 8.80 µM, respectively), demonstrating that bumetanide analogue **9c** was less toxic to human normal cells whilst effectively maintaining selectivity in the cancer cells. Compound **9c** is considered a promising anticancer candidate that requires further biological evaluation because it exhibits significant cell cytotoxic activity down to the one-digit micromolar IC_50_.

#### 2.1.3. Cell Cycle Analysis and Apoptosis Rate

**Figure 4:**
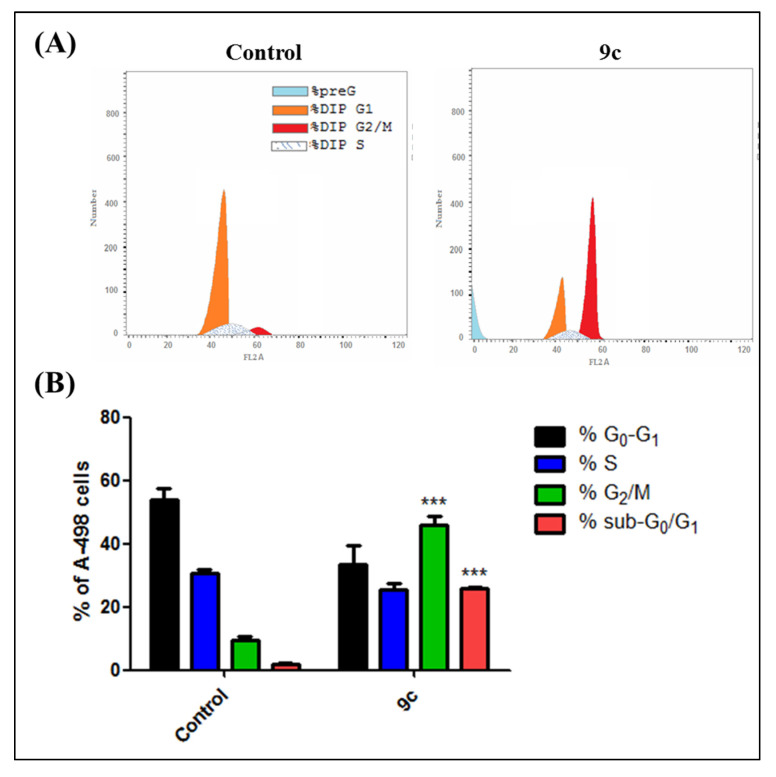
(**A**) Effect of compound **9c** on the cell cycle and apoptosis in kidney carcinoma cell line A-498. Cells were treated with either vehicle [0.1% ethanol (v/v)], **9c** (6 µM; approximately IC_50_ value) for 48 h. Cells were then fixed, stained with propidium iodide (PI), and analyzed by flow cytometry. Cell cycle analysis was performed on histograms of gated counts per DNA area (FL2-A). (**B**) Quantitative analysis of 4N (G2/M), 2N (G0G1), > 2N (S), and < 2N (sub-G1). DNA content was determined with CellQuest software. Values represent the mean ± SEM for three independent experiments. Statistical analysis was performed using one-way ANOVA-Bonferroni post-hoc test (***, *p* < 0.001).

**Figure 5:**
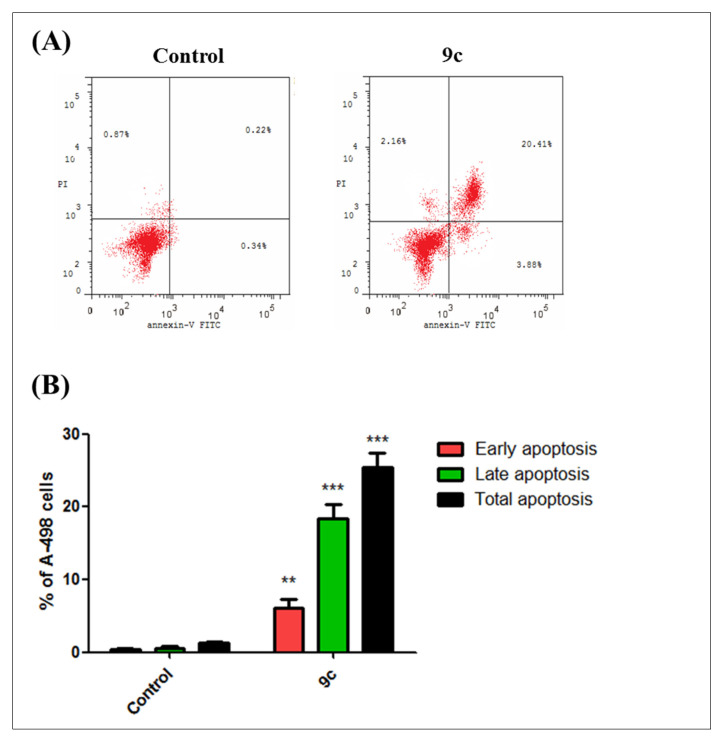
Compound **9c** potently induces apoptosis in kidney carcinoma cell line A-498 cells (Annexin V/PI FACS). (**A**) Effect of compound **9c** in A-498 cells analyzed by flow cytometry after double staining of the cells with Annexin-V-FITC and PI. A-498 cells treated with 6 µM (approximately IC_50_ value) of compound **9c** for 48 h and collected and processed for analysis. (**B**) Quantitative analysis of apoptosis. Values represent the mean ± SEM for three independent experiments. Statistical analysis was performed using one-way ANOVA-Bonferroni post-hoc test (**, *p* < 0.01; ***, *p* < 0.001). The four quadrants identified as: LL, viable; LR, early apoptotic; UR, late apoptotic; UL, necrotic.

The efficient antiproliferative activity of compound **9c** on the kidney carcinoma A-498 cell line (Table 2) prompted a further investigation of its mechanistic growth inhibitory action. Thus, the impact of bumetanide analog **9c** at 6 µM for 48 h on cell cycle distribution and induction of apoptosis in A-498 cells was next explored. As shown in Figure 4A, compound **9c** demonstrates that the predominant cell population at the G2/M stage (48.6%) is significantly more elevated than in the negative control untreated cell line (10.8%) (*p* < 0.001). Moreover, the number of cells in the sub-G1 phase was dramatically increased from 1.3% (control) to (25.5%) (*p* < 0.001) (Figure 4B). Analysis of apoptosis was performed to determine the mode of cell death induced by **9c** in A-498 cells. Compound **9c** induced both early and late apoptosis in A-498 cells at 6 µM when compared to the untreated control cells (*p* < 0.01 and *p* < 0.001, respectively) (Figure 5A). The average proportion of Annexin V-staining positive cells (total apoptotic cells) increased from 1.3% in control cells to 25.5% (*p* < 0.001) (Figure 5B). These findings are supported by flow cytometry findings above (Figure 4), which are in good agreement with literature, which reported **9c** to be a potent inhibitor of the *h*CA-IX isozyme. These results suggested that compound **9c** induces apoptosis of A-498 cells, which solidifies the anticancer potentiality of this promising drug candidate.

### 2.2. Computational Study

**Figure 6:**
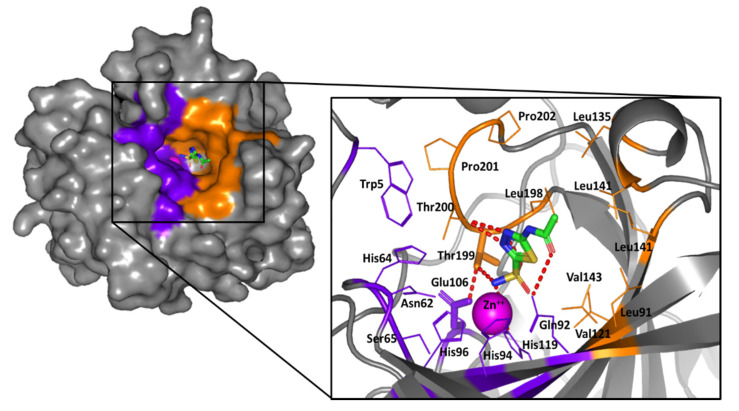
The surface rendition of *h*CA-IX (PDB ID: 3iai) in gray representation, illustrating the deeply anchored co-crystallized acetazolamide (sticks) into the active site cleft; the prosthetic Zn(II) (magenta sphere), as well as the hydrophilic (purple) and hydrophobic (orange) residues are shown. The zoomed image is the stereo view of acetazolamide (sticks; green as carbon, red as oxygen, blue as nitrogen, yellow as sulfur) occupying the chemically significant active site permitting tetrahedral coordination to Zn(II) and the histidine triad (His94, His96, His119). Hydrogen bonding is depicted as red dashed lines. Only residues located within 5Å radius of bound ligand are displayed as lines (except gate keepers-Thr199 and Glu106-as sticks) and labeled with sequence number.

Molecular docking studies were conducted for the representative ligands within the *h*CA-IX active site for enriched insights about the differential *h*CA-IX inhibition activities of the investigated compounds. Using the Molecular Operating Environment (MOE) Suite V.2014.09, the co-crystal structure of the *h*CA-IX as the biological drug-target (PDB ID: 3iai) was adopted within the docking workflow. This plasma membrane-associated enzyme is a two identical dimeric enzyme (Homo 2-mer-A2) bound to the clinically used sulfonamide inhibitor acetazolamide [43]. The crystallized ligand is categorized as classical *h*CA-inhibitor because it is deeply anchored into a 15 Å deep active site cleft with its primary deprotonated sulfonamide scaffold in coordination with the prosthetic zinc cation (ZnII) at the bottom of the active site. The N–Zn(II) adduct is further stabilized with three histidine residues (His94, His96, and His119, which are highly conserved across the 16 mammalian *h*CA isozymes), giving rise to the distinctive tetrahedrally-coordinative geometry (Figure 6) [18].

Notably, the ligand’s primary sulfonamide-based zinc-binding group (ZBG) interacts with one of the conserved residues, Thr199 and Glu106, which serve as the enzyme “gate keepers.” Serving fundamental roles within the catalysis process, Thr199 coordinates to the Zn(II)-bound water/hydroxide in the uninhibited enzyme, while Glu106 offers hydrogen bonding stabilization to Thr199 through its carboxylate moiety [18]. The periphery of the crystallized ligand is oriented at the center of the large open conical cavity spanning towards the surface of the protein. The canonical cavity shows two distinct halves made of hydrophobic or hydrophilic amino acids. Particularly, Asn62, His64, Ser65, Gln67, and Gln92 define the hydrophilic half of the active site, while Thr69, Leu91, Val121, Val131, Leu135, Leu141, Val143, Leu198, and Pro202 identify the lipophilic portion. The hydrophobic site division has the biological role of supporting the pathway for the entry and exit of both the natural substrate and product, while the hydrophilic face defines an ordered solvent network for proton transfer [43]. Notably, the establishment of favored contacts with the distal hydrophobic residues lining the active site entrance has been correlated to *h*CA-IX isozyme selectivity and defining the favored binding affinities of several *h*CA-IX inhibitors [37,50,51,52]. This selective pocket is located within the 5–15 Å radial zone, relative to the central position of the Zn(II)-ion, comprising the residues Thr69, Leu91, Val121, Val131, and Val143 [23,24,53].

The investigated compounds showed differential binding modes during virtual docking simulations, even though all ligands harbor the same primary sulfonamide pharmacophore as the classical ZBG. Few ligands managed to deeply anchor their ZBG within the conical cavity with proximity to the Zn(II)-ion depicting an orientation comparable with that of the crystallized ligand. At the same time, other ligands exhibited altered poses that are mostly retracted far from the prosthetic Zn(II)-ion with perfect occlusion of the catalytic site entrance. Such different binding modes could be explained by the substantial spatial differences of the ligands’ tails, which vary from being short, linear carbonyl-based synthons (**2** and **3**), to singly substituted heteroaromatic rings as in **4**, **5**, **6a**–**b**, and **9a**–**c**, or finally, the elongated hydrazone-linked chunky tails in ligands **7a**–**c**, and **8a**–**i**. The latter variations mediated the different establishment of viable contacts with either or both the hydrophilic and hydrophobic halves of the active site. The different binding modes among the ligands of the same chemical series are due to the electrostatic and steric features of different moieties.

**Figure 7:**
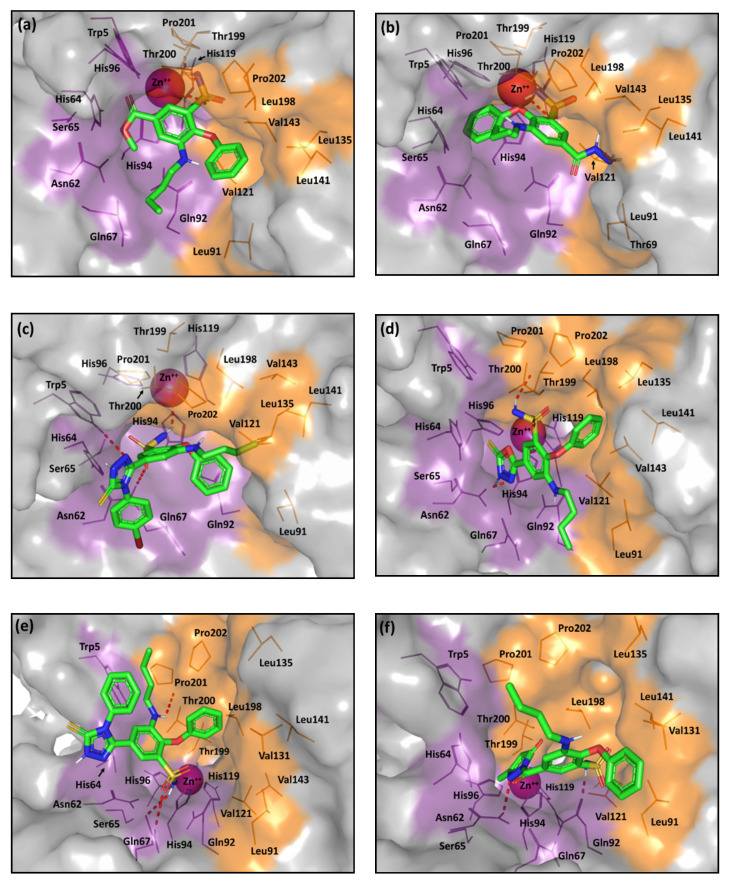
The predicted binding modes of the investigated compounds (sticks) at the *h*CA-IX binding site (PDB ID: 3iai); (**a**) **2**; (**b**) **3**; (**c**) **9c**; (**d**) **5**; (**e**) **9a**; (**f**) **4**. The active site cleft within the suggested ligand-protein complexes is illustrated as gray surface representation depicting the prosthetic Zn(II) (magenta sphere), as well as the hydrophilic (purple) and hydrophobic (orange) residues as lines. Hydrogen bonding is depicted as red dashed-lines, while polar coordination to Zn(II) as black dashed-lines. Only residues located within 5 Å radius of bound ligands are displayed (lines) and labeled with sequence number.

The small-sized tailed ligands anchored deeper within the active site cleft than the rest of the investigated compounds. Within this series, compounds **2** furnished significant docking scores with MOE *S* = −5.2332 Kcal/mol, respectively. As depicted, the phenoxy- and butylamino-functional groups within both compounds mediate favored support for the ligand-binding modes with relevant contacts with the hydrophilic and lipophilic site halves (Figure 7a). The sulfonamide group predicted coordination with the Zn ion as well as supporting polar interactions with the surrounding residues, Thr200 and the gate keeper Thr199. Similarly, ligand **3** exhibited comparable pose yet with single coordination between the sulfonamide and Zn ion. The lower binding energy assigned for the docked ligand **3** is suggested to an electrostatic penalty for the compound’s binding pose since its polar acylhydrazine moiety is directed towards the hydrophobic site division (Figure 7b). Nevertheless, hydrophobic (π-hydrogen) interaction with His64 managed to furnish a favored docking score of *S* = −5.0587 Kcal/mol (Supported information; Table S1).

Concerning the members of the intermediate-tailed series (**Series-I** and **-II**), their observed orientations and moderate molar ranged-activities suggested a prospective anchoring to the Zn(II)-coordinated solvated water molecule/hydroxide ion rather than direct formation of ligand-Zn(II) adducts.

Several ligands harboring the phenolic OH, primary amines, carboxylates, esters, or a simple sulfur atom have been reported for exhibiting such indirect anchoring to Zn(II)-ion [45,54,55,56,57,58,59,60,61,62,63,64].

The ZBGs of the intermediate-sized ligands with substituted single heterocyclic-ringed tails are retracted far from the prosthetic Zn(II)-ion while being directed towards the hydrophilic half of the site. Nevertheless, the heterocyclic-ringed arms play a significant role in directing both of the ligand’s hydrophobic moieties (phenoxy and butylamine functionalities) towards the hydrophobic residues of the selectivity pocket, furnishing single or multiple contacts with Leu91, Val121, Val131, or Val143. This finding is in agreement with the selective *h*CA-IX inhibitors introduced by Krasavin et al., where their 1,2,4-oxadiazol-5-yl ringed tails were not involved in any important direct interactions yet were essential for directing hydrophobic substitution (phenyl or pyridinyl ring) towards favored hydrophobic site interactions [65]. That result could be the reason why compounds **5** and **9b**,**c** (**Series-II**) exhibiting favored binding modes and one of the best docking scores (*S* = −5.1975, −6.4845 Kcal/mol, respectively).

The low nanomolar active ligands (**9b**,**c**) showed favored orientations for their triazole rings at the hydrophilic site division (supported π-proton interaction with Trp5; Supported information Table S1), causing both the allyl and *p*-bromophenyl substitutions to exhibit close proximity towards the His64 aromatic ring and furnishing favored π-proton interaction (Figure 7c). Moreover, extensive polar interactions with Asn62, Gln67, Thr200, and/or Trp5, allow these compounds to be well pinned within the CAIX active pocket. Comparable selectivity pocket-oriented binding mode was illustrated for compound 5 as an advent of its oxadiazole ring. The heterocyclic ring exhibited both polar support with Asn62/Thr200 and significant π-π stacking with His94 (Figure 7d). The common interaction for **Series-II** compounds and Asn62/Thr200 illustrates the significant role of such residues. At the same time, the counterparts, **4**, **6b**, and **9a** suggested a significant loss of the hydrophobic interface with the selectivity pocket residues. Figure 7e shows compound **9a** with an altered conformation of its triazole ring, the binding mode permits its phenyl substitution to be exposed to solvent far from His64 interface as well as lose the Asn62/Thr200 pinned interaction. Nevertheless, the extensive polar interactions with Gln67 may compensate the lost polar contacts and rationalize the compound’s one-digit nanomolar activity. On the other hand, unfavored orientations towards the selectivity pocket are illustrated for both ligands **4** and **6b** due to their acyl-extended, sterically restrained heteroaromatic scaffolds (Figure 7f). Moreover, an electrostatic penalty is predicted for these latter ligands having their ZBGs anchored too close to the lipophilic portion of the site.

**Figure 8:**
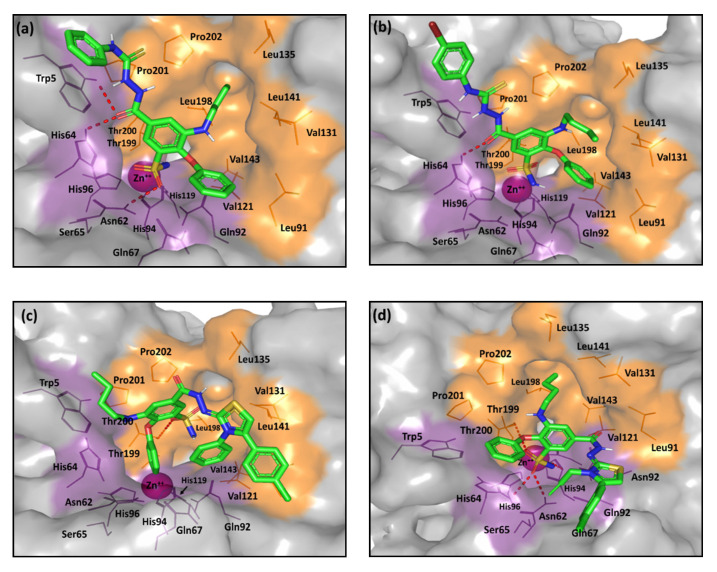
The predicted binding modes of the investigated compounds (sticks) at the *h*CA-IX binding site (PDB ID: 3iai); (**a**) **7a**; (**b**) **7c**; (**c**) **8a**; (**d**) **8d**. The active site cleft within the suggested ligand-protein complexes is illustrated as gray surface representation depicting the prosthetic Zn(II) (magenta sphere), as well as the hydrophilic (purple) and hydrophobic (orange) residues as lines. Hydrogen bonding is depicted as red dashed-lines, while polar coordination to Zn(II) as black dashed-lines. Only residues located within 5Å radius of bound ligands are displayed (lines) and labeled with sequence number.

The more extended-tailed ligands (**Series-III** and **-IV**) exhibited the most different docking poses compared to our short and intermediate-sized ligands. These large ligands possess a hydrophilic hydrazone-based linear or cyclic spacer between the central benzene scaffold and a terminal, branched, hydrophobic tailed part. These extended ligands were much more retracted towards the active site entrance, as they are highly branched. Such orientation permits an extended proper occlusion of the canonical catalytic site entrance against natural substrates (Figure 8). Several compounds have been reported within literature, to adopt this kind of occlusion binding while possessing significant *h*CA inhibitory activities. Occluding ligands that represent a diverse structural scaffold include coumarins, the anti-epileptic drug lacosamide, five- and six-membered lactones, thiolactones, or quinolinones [66,67,68]. Ligands with occlusion-binding modes exhibited lower *h*CA inhibition properties compared with the classical Zn(II)-binders. For this reason, our occluding ligands were presented with moderate inhibition activities of two-digit nanomolar IC_50_ values.

Notably, two distinct binding modes were predicted for the extended hydrazone-based derivatives, being at an inverse representation to each other. The respective poses of the micromolar active **7a**–**c** compounds (**Series-III**) predict the hydrazone-based scaffolds will be at the hydrophilic pocket side while the *meta*-phenoxy ring will be oriented towards the selectivity pocket (Figure 8a,b). However, being highly elongated, the terminal phenyl groups on the hydrazone-based tails are completely solvent exposed. Such pose could furnish an electrostatic penalty that could not be compensated through the supported hydrophobic/polar interaction with the Trp5 or His64 residues. At the same time, an inverse orientation is depicted for the **8a**–**i** ligands (**Series-IV**) where the more sterically hindered hydrazone-based extremities are directed to the hydrophobic site portion with their ZBG at close proximity to Gln67 or Gln92 lining the polar pocket half. This adopted orientation is favored for the steric hindrances of Trp5 and His64, making the hydrophilic site half more occluded, in addition to the increased hydrophobicity possessed by the terminal parts of the ligand tails. Interestingly, the ligands’ sulfonamide group affords their own fixation through several polar interactions. By exploring the structural activity relationship (SAR) of **8a**–**i**, different hydrophobicity profiles have been suggested for these ligands related to variable terminal substitutions, which range from the highly hydrophobic bromide derivatives to the least hydrophobic methoxy analogues. Among the favored hydrophobicity profiles, ligands as **8a** and **8h** exhibit the favored contacts with the hydrophobic residues at the selectivity pocket (Figure 8c). In contrast, **8d** showed an inferior hydrophobic profile because it harbors the allyl substituent instead of the more lipophilic phenyl moiety (Figure 8d). Nevertheless, the extensive polar interactions suggest favorable fixation and site occlusion for ligand **8d** which can interpret its one-digit nanomolar activity.

## 3. Materials and Methods

### 3.1. Carbonic Anhydrase Inhibition Assay

For assessing the inhibition of both target *h*CA isozymes II and IX, both the colorimetric Carbonic Anhydrase-Inhibitor^®^ Screening Kit (Cat. №. K473-100; BioVision^TM^, Milpitas, CA, USA) and ROBONIK-P2000^®^ ELISA microplate reader (Bio-Tek^TM^, Winooski, VT, USA) have been utilized. The kit-supplied carbonic anhydrase enzyme (BioVision^TM^, CA Enzyme) and purchased Human Recombinant *C*-terminal His-Tagged CA9 (Cat. №. 71101; BPS Bioscience^TM^, San Jose, CA, USA) have been applied for assessing the *h*CA-II and -IX inhibition, respectively. Based on the kit manufacturer’s protocol, the candidate inhibitors were dissolved in DMSO at 10 × highest final test concentration and specific aliquots of such working dilutions were incubated with the target *h*CA isozyme for 10 min at room temperature prior to permit the formation of the enzyme-inhibitor complex. Triplicate experiments were performed for each candidate inhibitor concentration. Parallel wells of solvent and enzyme controls were prepared to test the solvent impact upon the activity of the target enzyme. Furthermore, each candidate inhibitor had to be run alongside with its own background control where no proteins were added in such later wells. Following incubation, the CA-substrate was added and changes in absorbance (at 405 nm) were monitored for 60 min at room temperature within the kinetic mode. The negative enzyme control wells (without added compounds) were arbitrarily set as 100% enzyme activity. The mean absorbance for DMSO was negligible (A = 0.008). Consequently, the % inhibition of DMSO was insignificant, compared to the negative control. Absorbance readings from background wells were subtracted from the compound assay wells in order to account for background hydrolysis activity caused by the buffer solution [44,45]. The IC_50_ values were estimated through linear regression between the logarithm-transformed compound concentrations and corresponding % relative inhibitions = [(enzyme control Absorbance − compound Absorbance)/enzyme controlΔ Absorbance]* 100, having the slope as (ΔAbsorbance/Δt) for all samples.

### 3.2. MTT Cytotoxicity Assay

The colorimetric MTT Cell Proliferation^®^ Assay Kit (Cat. №. 10009365; Cayman Chemical^TM^, Ann Arbor, MI, USA) has been utilized to assess the cytotoxic impact of the investigated compounds on solid cancer cell lines [69]. The cancer cell lines, A-498-ATCC^®^ HTB-44^™^ kidney carcinoma, SCaBER- ATCC^®^ HTB-3™ bladder squamous cell carcinoma, and HEK-293 normal human embryonic kidney were obtained from American Type Culture Collection. Cells were cultured in 96-well plate, at cell density of 1.2–1.8 × 10^4^ cells/well, using Dulbecco’s Modified Eagle Medium (Invitrogen^TM^, Carlsbad, CA, USA) supplemented with 10% (w/v) fetal bovine serum (Hyclone^TM^, Marlborough, MA, USA), 10 ug/mL of insulin (Sigma-Aldrich^TM^, St. Louis, MO, USA), and 1% (w/v) penicillin-streptomycin (Sigma-Aldrich^TM^, St. Louis, MO, USA). Following 24h-incubation period under 5% CO_2_ at 37 °C, cells were treated with compounds’ serial concentrations (0.1 µM–100 µM) in DMSO, where the DMSO final concentration in the culture medium never exceeded 0.2% (v/v). Subsequently, the treated microplates were incubated for 48 h at 5% CO_2_/37 °C prior to examination under the inverted microscope and proceeding for the MTT assay. The culture medium was discarded and 100 µL of complete medium comprising 5 mg/mL MTT dye were dispensed within every well, then plates were reincubated for 2 h at 5% CO_2_/37 °C. Following a complete incubation, dissolution of the resulting formazan crystals was achieved through adding an amount of MTT Solubilization Solution [M-8910] equal to the original culture medium volume. Color intensity within each well was measured spectrophotometrically using the ROBONIK-P2000^®^ ELISA microplate reader (Bio-Tek^TM^, Winooski, VT, USA) at 450 nm while subtracting the background absorbance of multi well plates at 690 nm. The experiment was conducted in triplicates and IC_50_ values were estimated through linear regression between the logarithm-transformed compound concentrations and corresponding % cell viability = (mean absorbance in test wells/mean absorbance in control wells) ∗ 100, having the response variable slope (four parameters) for all samples.

### 3.3. Cell Cycle Analysis and Apoptosis Rate Investigation

A-498 Cells were seeded at a density of 1 × 10^5^ cells/well in 24-well plates and treated with compound **9c** (6 µM) for 48 h. The cells were collected by trypsinization and centrifuged at 800× *g* for 15 min. Cells were washed twice with ice-cold PBS and fixed in ice-cold 70% ethanol overnight at −20 °C. Fixed cells were centrifuged at 800× *g* for 15 min and stained with 50 μg/mL of PI, containing 50 μg/mL of DNase-free RNase A, at 37 °C for 30 min. The DNA content of cells (10,000 cells/experimental group) was analyzed by flow cytometer at 488 nm using a FACSCalibur flow cytometer (BD Biosciences, San Jose, CA, USA) and all data were recorded and analyzed using the CellQuest Software (Becton-Dickinson Biosciences, San Jose, CA, USA). For Annexin V/PI Apoptotic Assay, apoptotic cell death was detected by flow cytometry using Annexin-V/FITC Apoptosis Detection Kit (Cat. №. K101-25; Biovision^TM^, USA). Based on the kit manufacturer’s protocol, A-498 Cells were seeded in 24-well plated at density of 1 × 10^5^cells/mL and incubated for 24 h and subsequently treated with either vehicle (0.1% (v/v) EtOH), compound **9c** at 6 µM for 48 h. Cells were then harvested and prepared for flow cytometric analysis. Cells were washed in 1× binding buffer (20× binding buffer: 0.1 M HEPES, pH 7.4; 1.4 M NaCl; 25 mM CaCl2diluted in dH2O) and incubated in the dark for 30 min on ice in Annexin V-containing binding buffer [1:100]. Cells were then washed once in binding buffer and then re-suspended in PI-containing binding buffer [1:1000]. Samples were analyzed immediately using the BDaccuri flow cytometer (excitation = 488 nm; emission = 530 nm) and FCSExpress^®^ V.5 (De Novo Software^TM^, Pasadena, CA, USA). For analysis of the data, four populations are produced during the assay Annexin V and PI negative (Q4, healthy cells), Annexin V positive and PI negative (Q3, early apoptosis), Annexin V and PI positive (Q2, late apoptosis), and Annexin V negative and PI positive (Q1, necrosis).

### 3.4. Statistical Analysis

The analysis and visualization of data as well as comparative mean difference analysis was performed via the Prism^®^ Version 8 software (GraphPad^TM^, San Diego, CA, USA). Outcomes were expressed as mean ± SEM of three replicates (n = 3). Statistically significant differences between the afforded IC_50_ value of each compound as compared to control was estimated through student *t*-test. However, the mean differences between **9c** data versus the controls across the four cell cycles and two apoptosis stages and total, was estimated through one-way ANOVA Bonferroni post-hoc testing to check all possible pairwise combinations while not inflating the Type-1 error. Differences between groups were considered to be significant at a *p* value of < 0.05, ** *p* < 0.01, *** or *p* < 0.001.

### 3.5. Molecular Docking Protocol

The 21 investigated compounds were constructed using the builder interface within the MOE Suite software assigned for the current molecular docking studies. Energy-minimization in gas state for optimizing the ligand’s geometry was performed using Merck Molecular Force Field 94X (MMFF94X) and partial charges being automatically-estimated [70]. The furnished minimized ligands were then saved as Molecular Data Base (MDB) chemical file to be implemented within the further ligand-protein docking investigations. The X-ray crystal structure of the human membrane-bound carbonic anhydrase isozyme-IX (*h*CA-IX, PDB ID: 3iai) was set as the biological drug target for our virtual studies [19]. Obtained from the RCSB-Protein data bank, the proteinaceous tetrameric drug-target was loaded into the MOE software, prepared through 3D-protonation as well as auto-corrected for atoms connections, type, and charges. The binding site was defined by MOE Alpha Site Finder selecting the site involving the prosthetic zinc ion as well as the catalytic and canonical residues of the enzyme’s active site being reported within the literature. Dummy atoms were created from the obtained alpha spheres.

The default MOE rigid docking protocol was adopted where both the ligands and protein were set to be fixed. The rational for conducting such rigid-based docking studies, is that the calculated root mean square deviation (RMSD) between the binding site α-carbon atoms of *h*CA-IX both in apo- and holo-states (4zao and 3iai, respectively) was 0.80 Å. For having values below 1.0 Å, the calculated RMSD suggests that the three-dimensional topology of *h*CA-IX binding site within its holo-state furnishes a non-presentable difference when compared to its apo-state. Therefore, the local ligand induced-fit is suggested to have non-relevant impact on the *h*CA-IX binary complex structure, at least within the macromolecule crystalline state. Throughout the performed MOE rigid docking protocol, ligand conformations were generated with the bond rotation method, placed in the site with the triangle matcher method, and ranked with the London dG scoring function. The retained number of poses (pre-determined for 10 poses) were subjected to energy minimization within the pocket through the refinement process. Afterwards, a rescoring with the Generalized Born solvation model/Weighted Surface Area (GBVI/WSA) dG scoring function was conducted. PyMol v2.0.6 was used for analyzing and visually investigating the ligand-protein interactions of the obtained docking poses.

## 4. Conclusions

The study showed promising anticancer activity for the bumetanide-based analogues through targeting the tumor-associated hCA-IX enzyme. These compounds displayed potent and selective inhibitory activity over the cytosolic off-target hCA-II isoform with excellent selectivity indices (SI = 14.5–804) against the most abundant and catalytically reactive isozyme, *h*CA-II. Both **Series I** and **II** exhibit better inhibitory profile towards the tumor-associated hCA-IX isozyme than the bulkier-tailed compounds in **Series III**. However, the hCA-IX isoform was found to tolerate the bulky-tailed compounds in **Series IV**. An explanation for these different activities was successfully achieved through molecular docking studies, where sulfonamide-head anchoring, tail-part orientation, and favored ligand-residue contacts were investigated. Good correlation was found between the IC_50_ values and the docking scores, indicating that the docking results can be valuable tools for the prediction of the affinity of new hCA-IX blockers. The new derivative 9c displayed pronounced hCA-IX inhibitory activity and impressive antiproliferative activity on oncogenic A-498 cells and is considered to be a promising anticancer candidate. Future studies will optimize this compound, fine-tune its anticancer activity, and explore its potential through in-vivo preclinical studies.

## Figures and Tables

**Table 1:** In-vitro ^a^ data of the human carbonic anhydrase inhibition assay for the bumetanide compounds.

Compound	*h*CA-II IC_50_(nM) ^b^	*h*CA-IX IC_50_(nM) ^b^	Selectivity Ratio ^c^ II/IX
**2**	668.15	23.73	28.1
**3**	1614.82	7.87	205.1
**4**	1052.55	16.45	63.9
**5**	486.01	12.44	39.0
**6a**	N/D	16.56	
**6b**	858.26	5.43	158.9
**7a**	1571.85	17.74	88.6
**7b**	1344.25	14.39	93.4
**7c**	1535.37	12.91	118.9
**8a**	251.25	6.53	38.6
**8b**	1318.38	12.97	101.6
**8c**	473.66	12.60	37.5
**8d**	5117.31	6.36	804.6
**8e**	517.66	11.51	44.9
**8f**	999.18	13.41	74.5
**8g**	784.08	7.18	109.2
**8h**	208.81	14.31	14.5
**8i**	426.45	10.57	40.3
**9a**	4061.34	5.31	764.8
**9b**	205.21	7.25	28.3
**9c**	320.08	4.42	72.4
Acetazolamide	17.1	31.6	0.5

^a^ In-vitro data are reported from three replicate experiments. Statistical significance set at *p* < 0.05 compared to reference standard. ^b^ IC_50_ values represent the effective concentration of a given compound for exhibiting 50% inhibitory response of that compound’s intrinsic maximum response. N/D; denotes non-detected IC_50_ within the range of the tested concentrations. ^c^ Selectivity as determined by the ratio of IC_50_ values for isozyme CA-II relative to CA-IX.

**Table 2:** In-vitro ^a^ data of the MTT cell proliferation assay for the bumetanide compounds.

Compound	A-498 IC_50_ (μM) ^b^	SCaBER IC_50_ (μM) ^b^
Acetazolamide	40.00 ± 0.37	40.63 ± 0.36
**8a**	15.66 ± 0.63	28.36 ± 1.4
**8h**	6.55 ± 0.26	78.04 ± 0.29
**9b**	16.08 ± 1.4	78.07 ± 1.7
**9c**	5.07 ± 0.11	8.80 ± 1.9

^a^ In-vitro data are reported from three replicate experiments ±SEM. ^b^ IC_50_ values represent the effective concentration of a given compound for exhibiting 50% inhibitory response of that compound’s intrinsic maximum response.

## Supplementary Materials

The following are available online at https://www.mdpi.com/1424-8247/13/9/252/s1, Table S1: Data of the virtula docking simulations conducted for the bumetanide compounds against the human carbonic anhydrase isozyme-type IX.
