# Supplementary material for: The Anticancer Activity for the Bumetanide-Based Analogs via Targeting the Tumor-Associated Membrane-Bound Human Carbonic Anhydrase-IX Enzyme
Source: Pharmaceuticals (Basel). 2020 Sep 18;13(9):252. doi: 10.3390/ph13090252 (PMC7558282; doi:10.3390/ph13090252)

Table S1: Data of the virtula docking simulations conducted for the bumetanide compounds against the human carbonic anhydrase isozyme-type IX

<table>
<tr><th>Docked Ligand</th><th>MOE (S) [a]
(Kcal/moL)</th><th>RMSD [b]
(Å)</th><th>Ligand-target interaction descriptions [c]
[Type of Interactions; Length (Å); Angle (°); Interacting Residues]</th></tr>
<tr><td>Crystallized Ligand (Redocked)</td><td>-2.1678</td><td>2.07</td><td>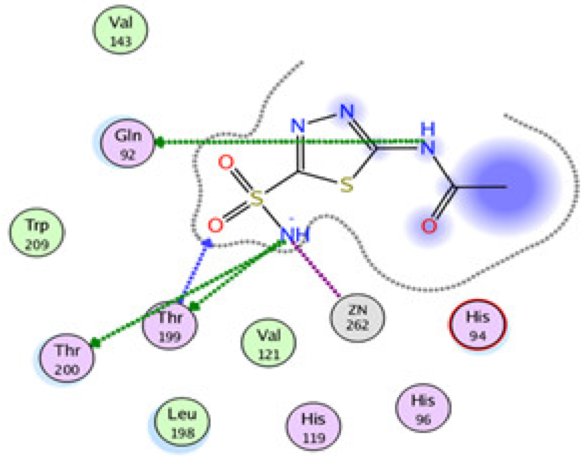

Coordination ; 2.9 Å ; ZnII prosthetic group
H-bonding ; 2.4 Å ; 110.7° ; Thr-200 (Side chain –OH)
H-bonding ; 1.9 Å ; 140.5° ; Thr-199 (Side chain –OH)
H-bonding ; 2.0 Å ; 146.6° ; Thr-199 (Side chain –OH)
H-bonding ; 2.5 Å ; 120° ; Gln-92 (Side chain –C(O)NH–)</td></tr>
<tr><td>Ligand 2</td><td>-5.2332</td><td>1.99</td><td>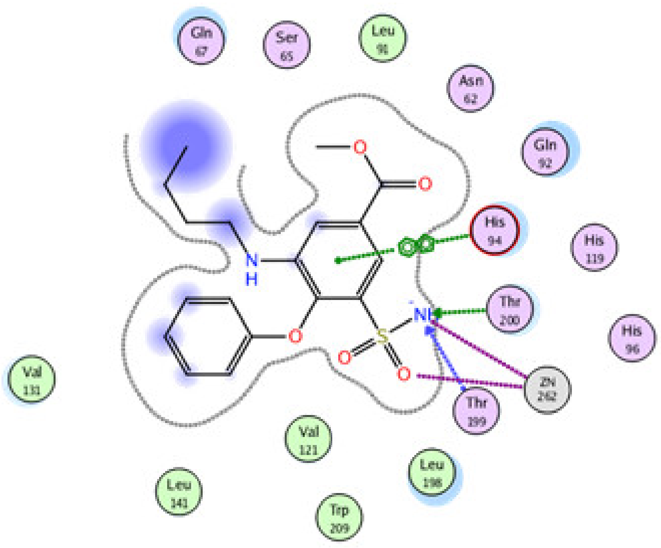

Coordination ; 3.7 Å ; ZnII prosthetic group
Coordination ; 3.2 Å ; ZnII prosthetic group
H-bonding ; 2.1 Å ; 110.7° ; Thr-199 (Side chain –OH)
H-bonding ; 2.2 Å ; 110.7° ; Thr-200 (Side chain –OH)
Arene-Arene interaction ; 2.6 Å ; His-94</td></tr>
</table>

**Ligand 3**	-5.0587	1.80	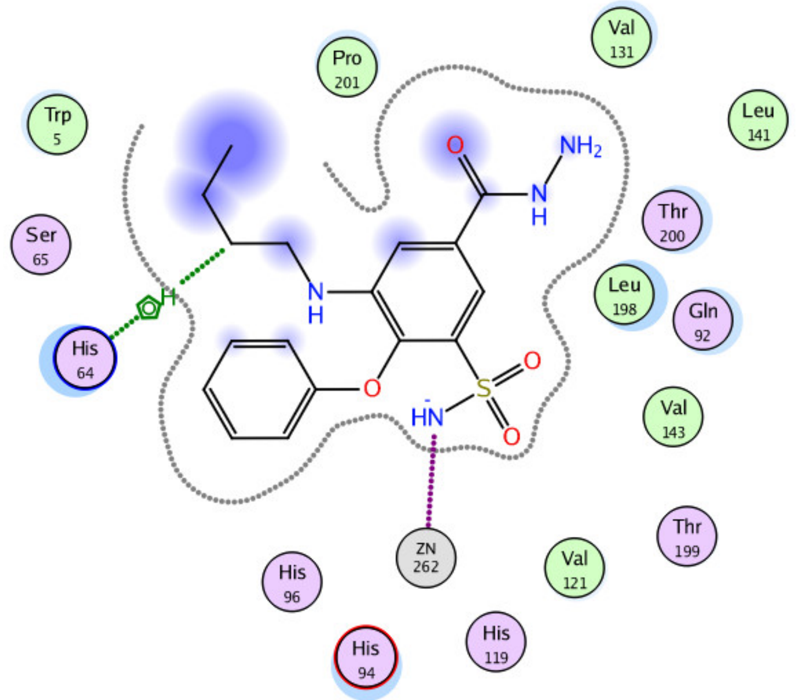<br>Coordination **;** 3.0 **Å ;** ZnII prosthetic group<br>Arene-Hydrogen interaction **;** 3.7 **Å ;** His-94
**Ligand 4**	-4.6697	3.09	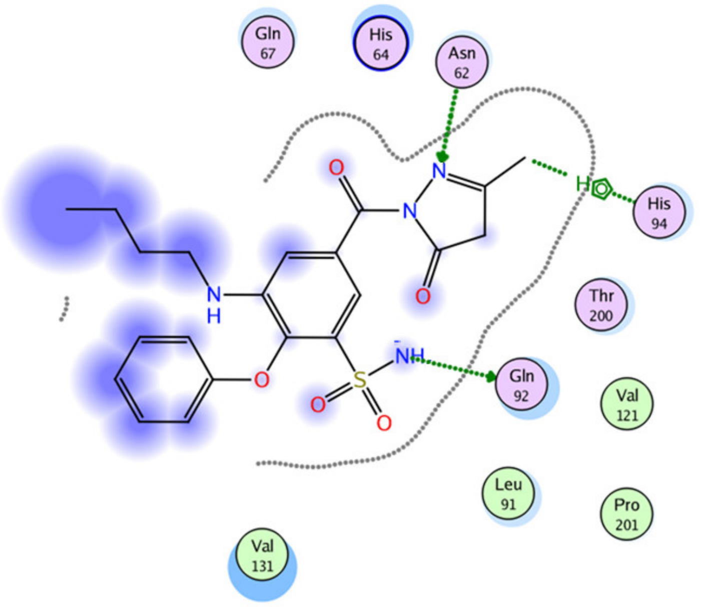<br>Hydrogen bonding **;** 2.5 **Å ;** 113.1° **;** Asn-62 (Side chain –NH**H**)<br>Hydrogen bonding **;** 1.9 **Å ;** 160.8° **;** Gln-92 (Side chain –**N**H_2)<br>Arene-Hydrogen interaction **;** 3.2 **Å ;** His-94

<table>
<tr><td>Ligand 5</td><td>-5.1975</td><td>2.36</td><td>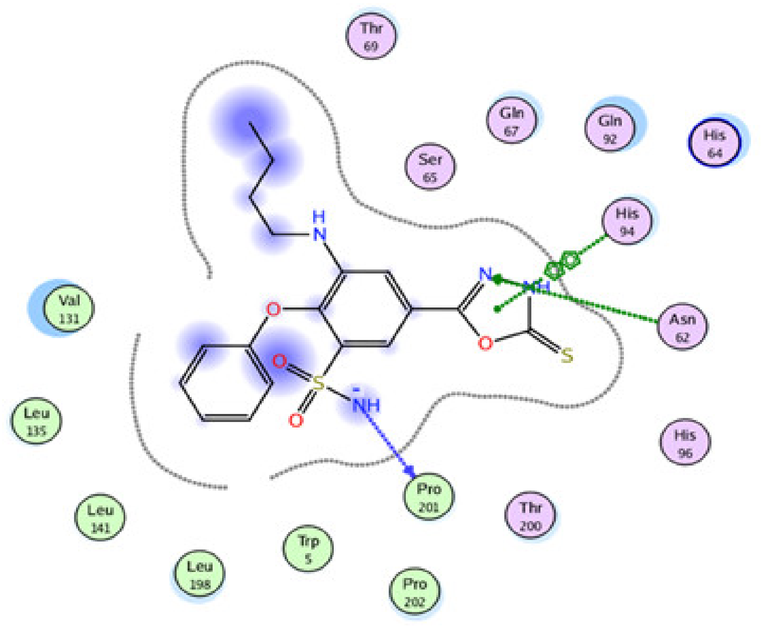

Hydrogen bonding ; 2.1 Å ; 135.2° ; Asn-62 (Side chain –NHH)
Hydrogen bonding ; 2.3 Å ; 148.9° ; Pro-201 (Main chain –C(O)NH–)
Arene-Arene interaction ; 3.2 Å ; His-94</td></tr>
<tr><td>Ligand 6a</td><td>-5.0228</td><td>1.64</td><td>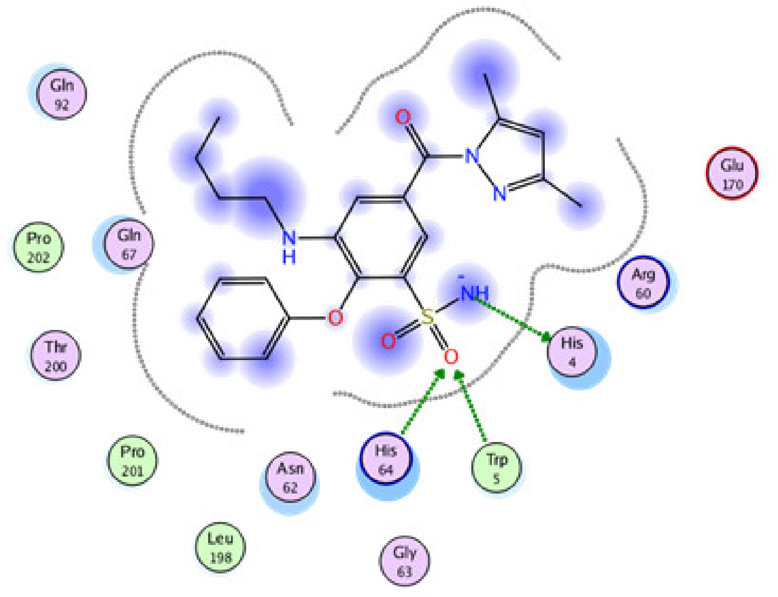

Hydrogen bonding ; 2.3 Å ; 159.8° ; His-4 (Side chain =N)
Hydrogen bonding ; 2.2 Å ; 134.2° ; His-64 (Side chain –NH)
Hydrogen bonding ; 2.4 Å ; 131.3° ; Trp-5 (Side chain –NH)</td></tr>
</table>

**Ligand 6b**	-4.8280	2.01	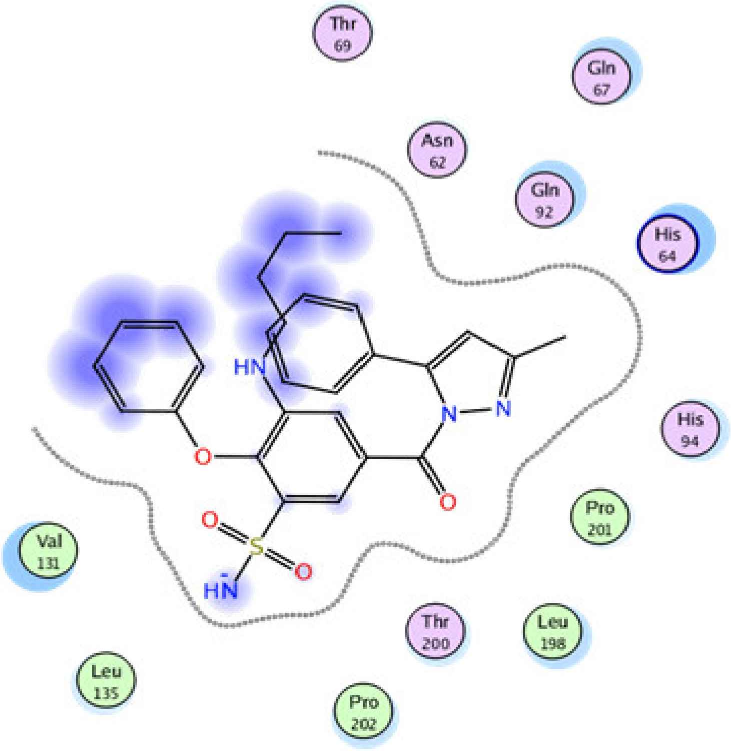
**Ligand 7a**	-6.2632	1.56	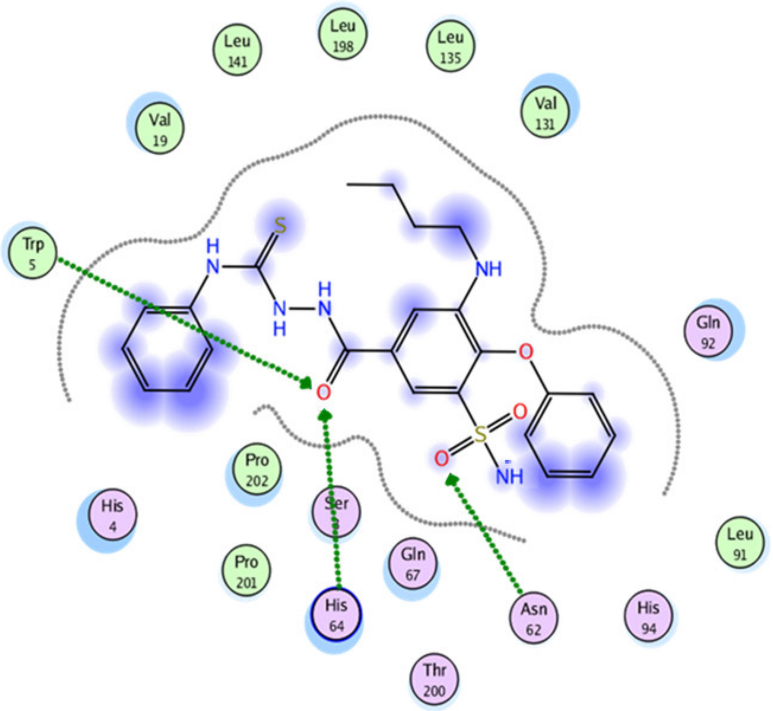<br>Hydrogen bonding ; 2.7 **Å** ; 107.1° ; Asn-62 (Side chain –NH**H**)<br>Hydrogen bonding ; 2.4 **Å** ; 108.2° ; His-64 (Side chain –N**H**)<br>Hydrogen bonding ; 3.2 **Å** ; 132.7° ; Trp-5 (Side chain –N**H**)

**Ligand 7b**	-5.2799	3.37	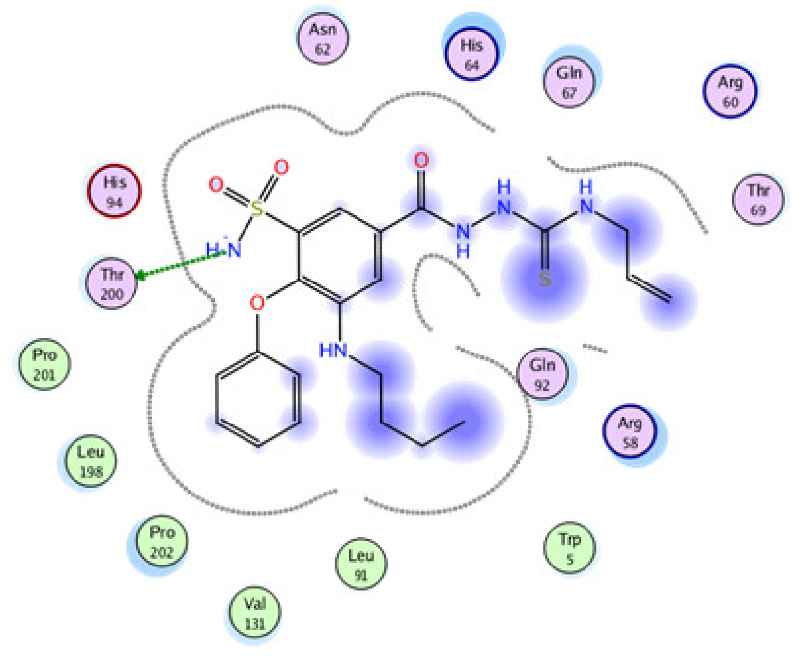<br>Hydrogen bonding ; 2.5 **Å** ; 137.4° ; Thr-200 (Side chain **–O**H)
**Ligand 7c**	-5.8080	1.81	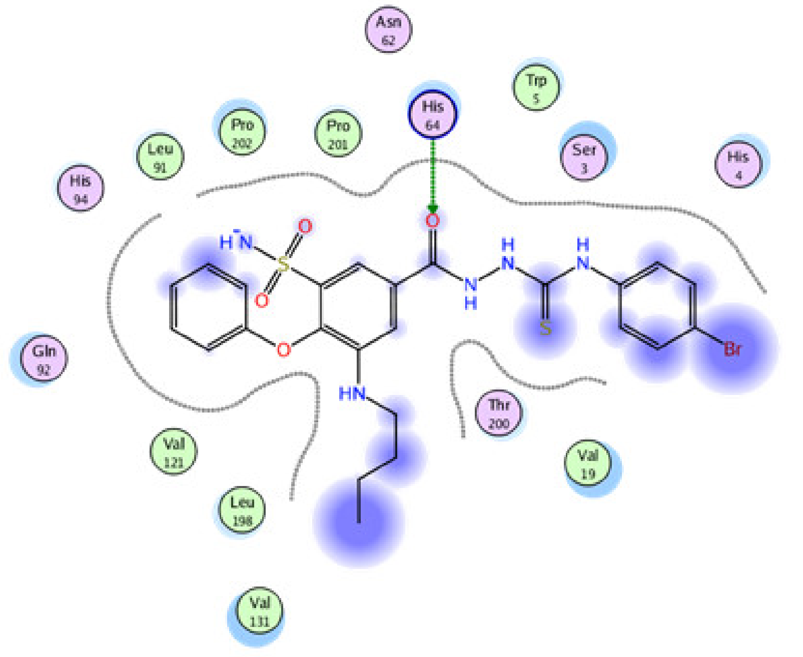<br>Hydrogen bonding ; 2.3 **Å** ; 119.3° ; His-64 (Side chain –N**H**)

**Ligand 8a**	-5.4913	3.08	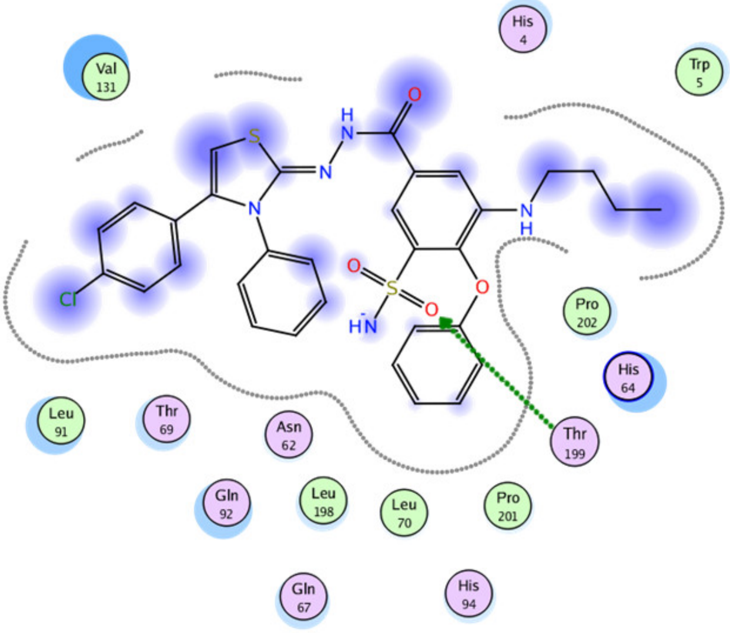<br>Hydrogen bonding ; 2.5 **Å** ; 139.4° ; Thr-199 (Side chain **–**N**H**)
**Ligand 8b**	-6.4351	2.91	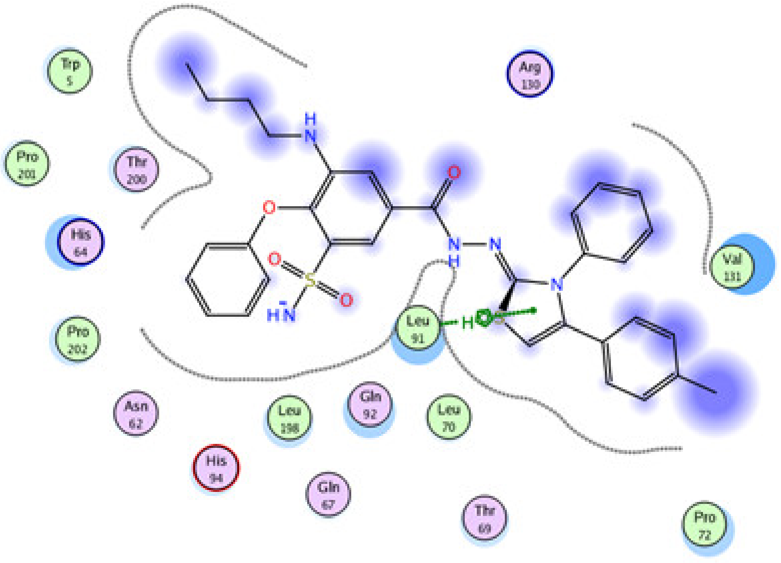<br>Arene-Hydrogen interaction ; 3.2 **Å** ; Leu-91 (Side chain **–**CH**H–**)

**Ligand 8c**	-4.8966	3.16	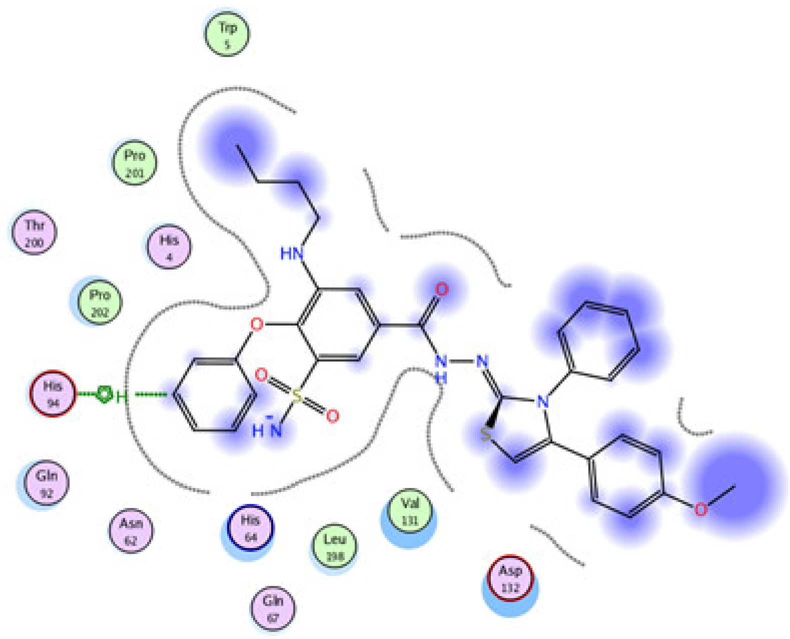<br>Arene-Hydrogen interaction ; 4.4 **Å** ; His-94
**Ligand 8d**	-6.2110	2.54	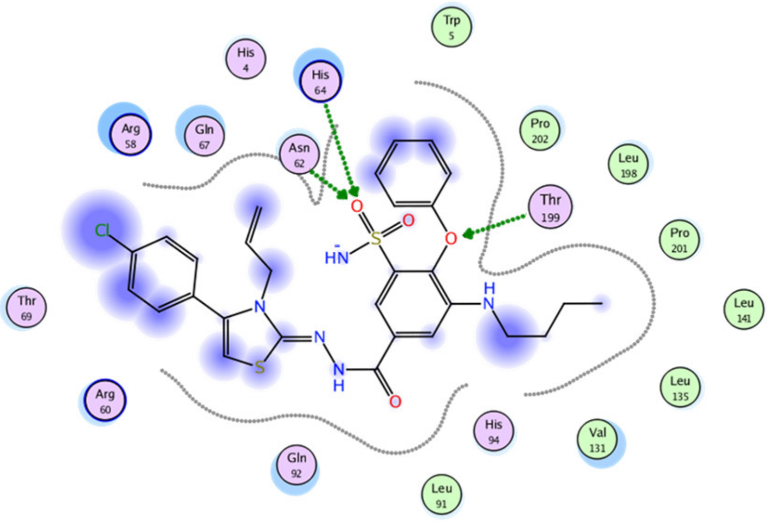<br>Hydrogen bonding ; 2.9 **Å** ; 129.4° ; His-64 (Side chain –N**H**)<br>Hydrogen bonding ; 3.1 **Å** ; 144.5° ; Asn-62 (Side chain –N**H**)<br>Hydrogen bonding ; 3.2 **Å** ; 122.5° ; Thr-199 (Side chain –O**H**)

**Ligand 8e**	-5.7674	2.77	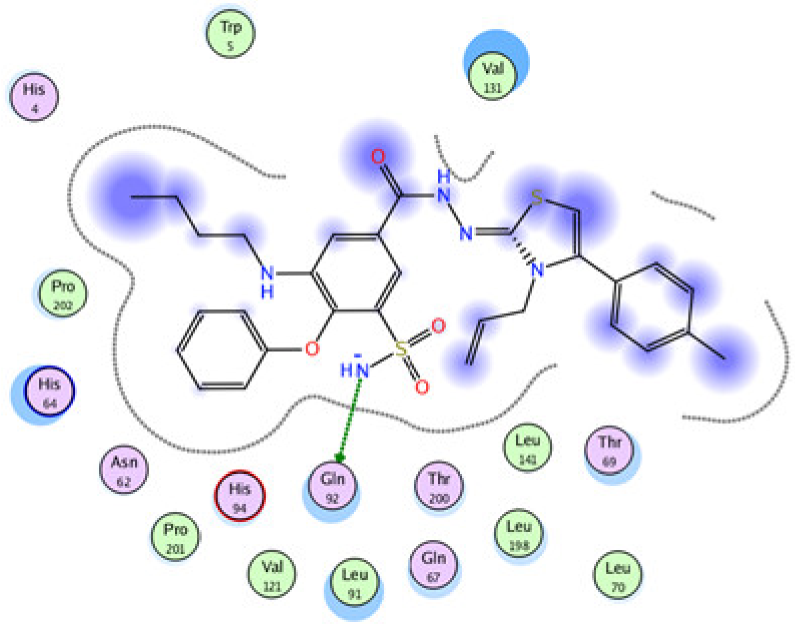<br>Hydrogen bonding ; 2.3 **Å** ; 146.5° ; Gln-92 (Side chain –C(**O**)NH–)
**Ligand 8f**	-5.1837	1.70	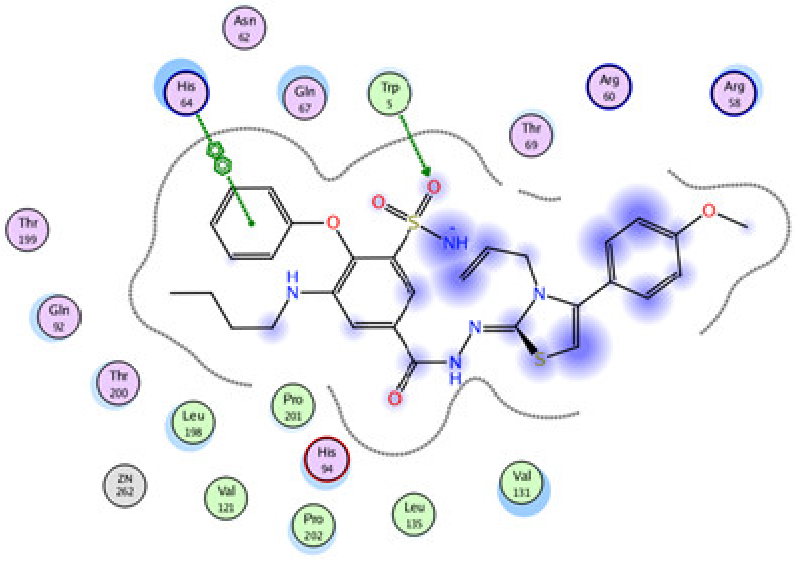<br>Hydrogen bonding ; 2.2 **Å** ; 152.6° ; Trp-5 (Side chain –N**H**)<br>Arene-Arene interaction ; 3.7 **Å** ; His-64

**Ligand 8g**	-6.2308	2.50	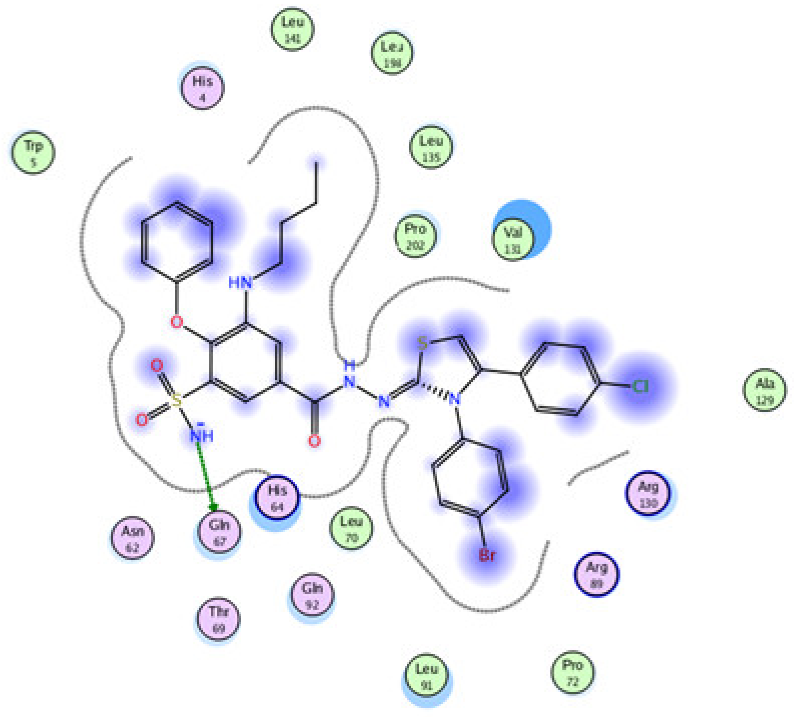<br>Hydrogen bonding ; 2.1 **Å** ; 153.0° ; Gln-67 (Side chain –C(**O**)NH–)
**Ligand 8h**	-5.4212	1.69	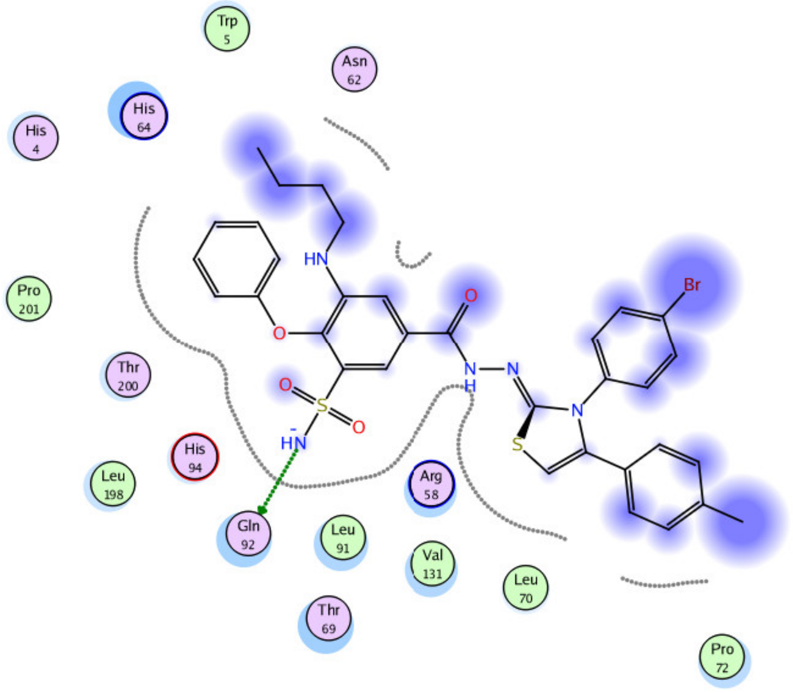<br>Hydrogen bonding ; 2.1 **Å** ; 144.0° ; Gln-92 (Side chain –C(**O**)NH–)

**Ligand 8i**	-4.9819	1.05	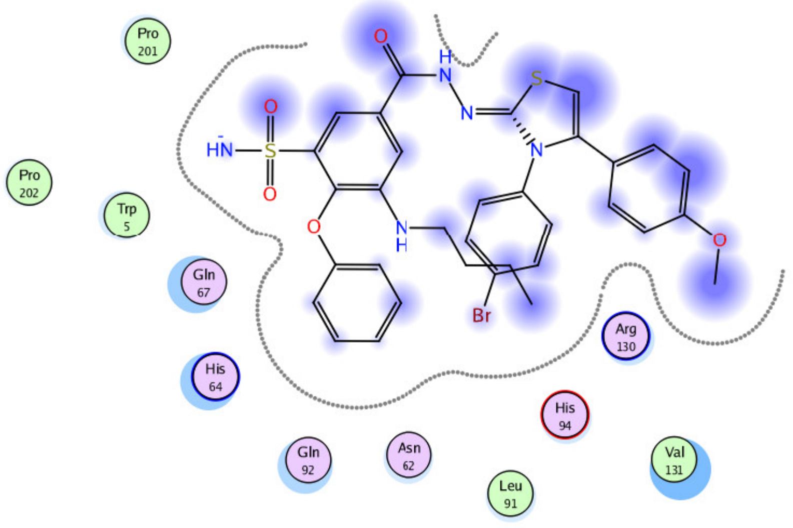
**Ligand 9a**	-4.3762	2.39	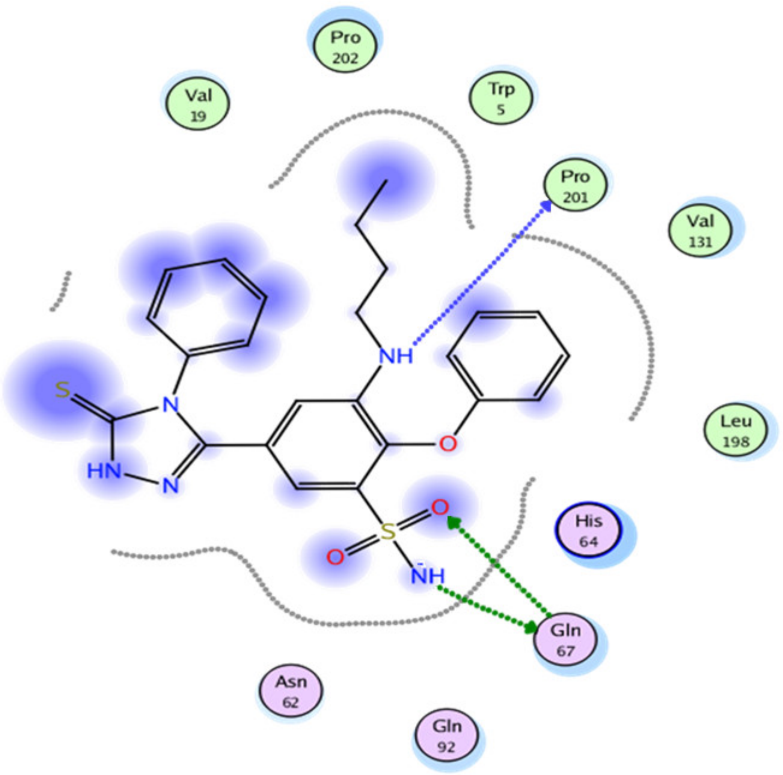<br>Hydrogen bonding ; 2.6 **Å** ; 141.0° ; Gln-67 (Side chain –C(**O**)NH_2)<br>Hydrogen bonding ; 2.9 **Å** ; 122.9° ; Gln-67 (Side chain –C(O)NH**H**)<br>Hydrogen bonding ; 3.0 **Å** ; 161.0° ; Pro-201 (Main chain –C(**O**)NH–)

**Ligand 9b**	-6.4845	1.94	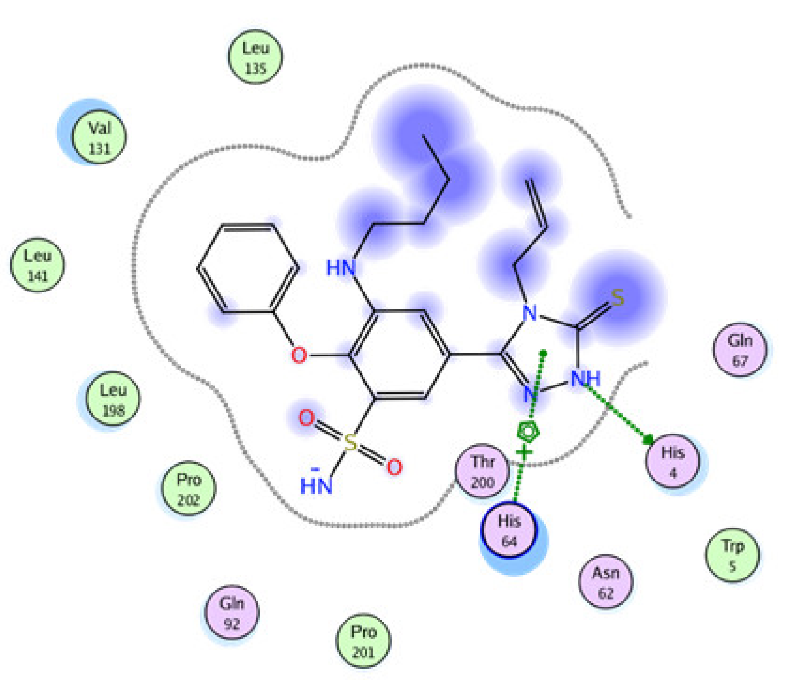<br>Hydrogen bonding ; 2.1 **Å** ; 147.7° ; His-4 (Side chain =**N**–)<br>Arene-Cation interaction ; 3.5 **Å** ; His-64 (Side chain –**N**$^{+}H_{2}$–)
**Ligand 9c**	-5.8355	1.63	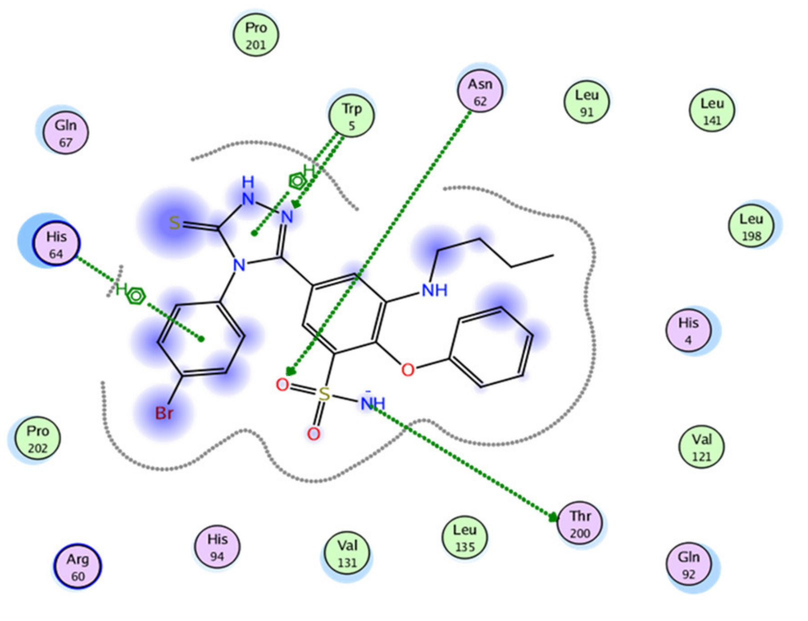<br>Hydrogen bonding ; 2.7 **Å** ; 129.6° ; Thr-200 (Side chain –**O**H)<br>Hydrogen bonding ; 3.0 **Å** ; 106.9° ; Asn-62 (Side chain –N**H**)<br>Hydrogen bonding ; 2.7 **Å** ; 124.**5°** ; Typ-5 (Side chain –N**H**)<br>Arene-Hydrogen interaction ; 3.2 **Å** ; Trp-5 (Side chain –N**H**)<br>Arene-Hydrogen interaction ; 3.0 **Å** ; His-64 (Side chain =C**H**–)

[a] MOE (***S***); Docking scores utilizing the scoring function assigned for the best-ranking poses which have been selected relying on the visual examination and after refinement through the rescoring function of the GBVI/WSA dG.

[b] RMSD; root mean square deviation of the best-ranking pose as compared to the crystal.

[c] The Legand for the predicted Ligand-enzyme residues interactions as follows:

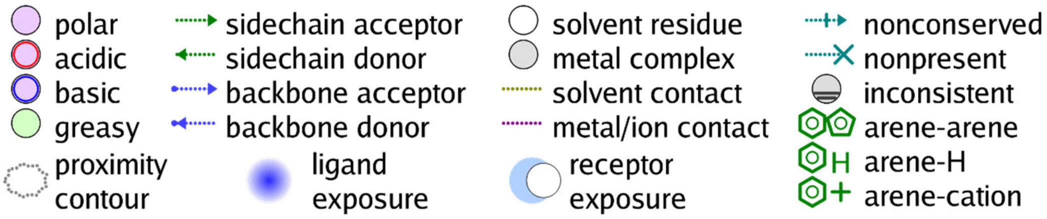

Supplement: Supplementary file 1 [file pharmaceuticals-13-00252-s001.pdf]
